# Developing an Improved UHPLC Method for Efficient Determination of European Pharmacopeia Process-Related Impurities in Ropinirole Hydrochloride Using Analytical Quality by Design Principles

**DOI:** 10.3390/molecules25112691

**Published:** 2020-06-10

**Authors:** Tim Tome, Aleš Obreza, Zdenko Časar

**Affiliations:** 1 Faculty of Pharmacy, University of Ljubljana, Aškerčeva c. 7, Ljubljana SI-1000, Slovenia; tim.tome@sandoz.com; 2 Analytics Department, Sandoz Development Center Slovenia, Lek Pharmaceuticals d.d., Verovškova ulica 57, Ljubljana SI-1526, Slovenia

**Keywords:** AQbD, UHPLC, method development, method optimization, design of experiments, pharmacopeia, ropinirole hydrochloride

## Abstract

This article presents the development of a reversed-phase ultra-high-performance liquid chromatographic method for determining process-related impurities in ropinirole hydrochloride drug substance applying the analytical quality by design approach. The current pharmacopeial method suffers from selectivity issues due to two coelutions of two pairs of impurities. The development of a new method began with preliminary experiments, based on which the Acquity UPLC BEH C8 was selected as the most appropriate column. The effects of six different critical method parameters (CMPs) were then investigated using a fractional factorial screening design. Column temperature, the ratio of methanol in mobile phase B, and gradient slope turned out to be highly significant CMPs in achieving critical resolutions, and they were further evaluated using a central composite face-centered response-surface design. Mathematical models were created by applying a multiple linear regression method. Based on the elution order of an unknown degradation impurity and impurity C, two design spaces were established, and for each design space an optimal combination of CMPs was determined. The method developed was validated for precision, accuracy, linearity, and sensitivity, and it was proven suitable for determining nine process-related impurities of ropinirole.

## 1. Introduction

**Figure 1:**
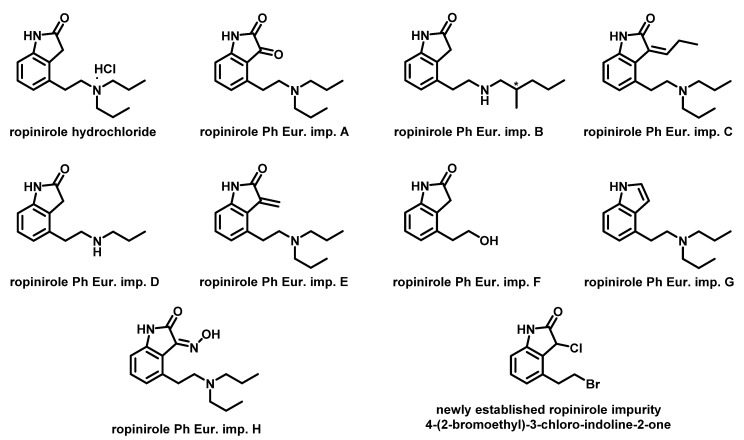
Structure of ropinirole hydrochloride, its process-related impurities defined in European Pharmacopeia [22] and the structure of 4-(2-bromoethyl)-3-chloro-indoline-2-one (chloro impurity).

Ropinirole hydrochloride (Figure 1) is a non-ergoline dopamine agonist indicated for the treatment of Parkinson’s disease [1,2,3,4] and moderate-to-severe primary restless legs syndrome [5,6,7]. Ropinirole was first approved in the United States by the Food and Drug Administration in 1997, and then in 2004 by European Medicines Agency. The worldwide market value of ropinirole in 2018 was USD 248 million according to IQVIA Analytics Link™ data. Recently, new investigations have indicated that ropinirole might also be a good drug candidate for the treatment of amyotrophic lateral sclerosis [8].

Several liquid chromatography–based analytical methods for determining ropinirole in drug substance and in final dosage forms have been reported [9,10,11,12,13,14]. Liquid chromatography methods for determining degradation products in ropinirole formulations are also documented in the literature [15,16]. In addition, a method for determining ropinirole and its metabolites in plasma is also known [17].

Surprisingly, although some liquid chromatography analytical methods covering process-related impurities of ropinirole exist in the literature, they mainly cover a very limited amount of known process-related impurities [18,19,20,21]. Indeed, the method of McArdle et al. covers only ropinirole Ph. Eur. impurity C [18]. Escott et al. described a high-pressure liquid chromatographic (HPLC) method using diode array detection (DAD) for monitoring Ph. Eur. impurity A in ropinirole [19]. Deshpande et al. isolated and separated Ph. Eur. impurity A, Ph. Eur. impurity D, and another process-related impurity (5-(2-(dipropylamino)ethyl)-1,4-dihydro-3*H*-benzo[*c*][1,2]oxazin-3-one) from ropinirole drug substance using the HPLC method [20]. Finally, Medenica et al. investigated the chromatographic behavior of ropinirole and its Ph. Eur. impurities A and D in the presence of inorganic salts [21]. This is in sharp contrast to European Pharmacopoeia (Ph. Eur.) monograph 2604 for ropinirole hydrochloride, which describes eight process-related impurities A–H of ropinirole (Figure 1) and provides chromatographic conditions for their separation [22]. Moreover, 4-(2-bromoethyl)-3-chloro-indoline-2-one (Figure 1), named chloro impurity further in the text, which is a late-stage intermediate in ropinirole synthesis [23,24], appeared as an additional process-related impurity of ropinirole that should be controlled in the drug substance.

Although so far Ph. Eur. monograph 2604 for ropinirole hydrochloride provides the most comprehensive list of key process-related impurities of ropinirole and describes chromatographic conditions for their separation, we had the experience that the compendial method does not separate impurities E and H of ropinirole. Additional coelution was observed between impurities B and G. Furthermore, the method has an extremely long run time of 75 min, which limits the throughput of industrial analyses. Finally, the chloro impurity mentioned above is not currently covered in Ph. Eur. monograph 2604 for ropinirole hydrochloride. All these facts provide a great impetus for developing a new liquid chromatography method that should fully separate all eight Ph. Eur. impurities of ropinirole hydrochloride and chloro impurity from drug substance and each other. In addition, the method should be significantly shorter compared to the current Ph. Eur. method.

This article describes the development and optimization of a selective and robust analytical method for determining nine process-related impurities of ropinirole hydrochloride (Figure 1). The development of a new method for determining ropinirole process-related impurities followed principles of analytical quality by design (AQbD) [25]. With the AQbD approach, improved method performance with a defined area of method robustness can be accomplished. Therefore, utilization of AQbD concepts in developing liquid chromatographic methods for determining either active pharmaceutical ingredients or their impurities have flourished in recent years, as evidenced by numerous reports on their successful application [26,27,28,29,30,31,32,33,34,35,36,37,38,39,40,41,42,43,44,45]. The use of AQbD concepts in liquid chromatography method development renders these methods more robust, makes possible their facile validation, and frequently provides shorter run times for the separation of the same number of analytes compared to methods developed using the one-factor-at-a-time (OFAT) approach [25]. In addition, a new International Council for Harmonization (ICH) Q14 guideline covering the topic of AQbD is expected in 2021 and will favor utilization of AQbD in method development [46]. Finally, the method developed for ropinirole hydrochloride has been validated in terms of precision, sensitivity, accuracy, and linearity.

## 2. Results and Discussion

### 2.1. Initial Testing of the Existing Ropinirole Ph. Eur. HPLC Method

**Figure 2:**
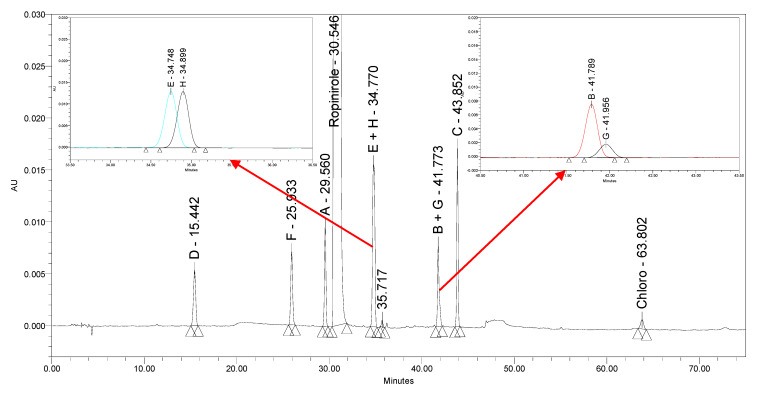
A chromatogram of ropinirole hydrochloride spiked with Ph. Eur. impurities A–H and chloro impurity obtained using the Ph. Eur. method. The co-elution between E and H is presented in the upper left corner, the co-elution between B and G is presented in the upper right corner.

The current Ph. Eur. method [22] employs reversed-phase (RP) HPLC separation on a C8 stationary phase (Kromasil C8 5 µm, 250 × 4.6 mm) using a gradient elution principle with acidic buffer (pH = 2.5) as mobile phase A and 70/30% (*v/v*) solution of acetonitrile/methanol as mobile phase B with UV detection at 250 nm within 75 min. While injecting Ph. Eur. impurities A–H and chloro impurity under the prescribed conditions, a lack of selectivity of the Ph. Eur. method was observed. Impurity E coeluted with impurity H at a retention time of approximately 35 min and impurity B coeluted with impurity G at a retention time of approximately 42 min. Figure 2 presents the chromatogram of ropinirole hydrochloride at a concentration of 2.0 mg/mL spiked with nine impurities investigated at a 0.2% level obtained using the Ph. Eur. method.

Due to the aforementioned coelution problems and because we intended to shorten the analysis time, we decided to develop a new method.

### 2.2. Analytical Target Profile

The first step in AQbD method development is the definition of the analytical target profile (ATP). The ATP includes the purpose of the analytical method and the measurement criteria that the method needs to be capable of fulfilling [47]. For the method under development, the ATP was the following: The method should be capable of quantitatively determining all nine process-related impurities of ropinirole in ropinirole hydrochloride from the reporting threshold to the specification limit prescribed in the Ph. Eur. monograph for ropinirole hydrochloride.

### 2.3. Method Scouting (Preliminary Experiments)

At the very beginning of preliminary experiments, we decided to use the ultra-high performance liquid chromatography (UHPLC) system because it may operate at higher pressures (up to 20,000 psi), compared to the HPLC system. Such an advantage allows the use of smaller particle size columns (sub-2 μm). UHPLC columns are shorter with a smaller diameter, resulting in a shorter analysis time.

We started with an overview of several C8 and C18 stationary phases with either solid-core (UPLC BEH C8, UPLC HSS T3, UPLC HSS C18 SB, and UPLC CSH C18 from Waters Corporation, Milford, MA, USA) or core-shell (CORTECS UPLC C8 from Waters and Kinetex C8 from Phenomenex, Torrance, CA, USA) particles, which we considered the most appropriate for separating the analytes, according to their chemical structures. A significantly better separation between impurity A and ropinirole was observed on a C8 column compared to C18, which is stated as the critical separation in the Ph. Eur. method. In addition, the tailing factor of the main peak was lower when using a solid-core particle column compared to core-shell, which significantly affected the critical separation, because impurity A eluted just before ropinirole. Based on those results, we selected the BEH C8 column.

Another critical separation was between impurities E and H. Most of the analytes investigated are basic molecules. According to their p*K_a_* curves (obtained with MarvinSketch, ChemAxon, Budapest, Hungary), they are more than 90% positively charged at a pH under 9.2 and have a p*K_a_* value around 10.1 to 10.5, except for impurity F, being non-ionized under a pH of 11.2. Thus, pH may significantly influence their separation. For this purpose, the effect of different pH ranges on the selectivity of the method was explored during method scouting: a pH of 2.5 and 7.7 using an ammonium phosphate buffer and a pH of 5.0 using an ammonium acetate buffer. In fact, the pH of 7.7 caused shifting (a different elution order) of some impurity peaks, including impurity H, resulting in significantly improved separation between impurities E and H. Higher pH also resulted in later elution of ropinirole itself with a lower tailing factor. However, at a pH of 7.7 impurity A eluted under the main peak of ropinirole. Moreover, using a pH of 5.0 or 7.7 resulted in distinct fronting of the impurity E peak, which limits its accurate quantitative determination. The acidic pH using a phosphate buffer turned out to be optimal.

Because ropinirole and its impurities are relatively small molecules (the molecular weight of ropinirole is 260.4), we considered buffer molarity to influence the peak shape of ropinirole. Indeed, higher buffer molarity caused a lower tailing factor, which contributed to better resolution between impurity A and ropinirole. During preliminary experiments, we also observed that the column temperature influences the separation between impurities E and H. However, other parameters were also included in the method performance, for which we did not know the effects: the ratio of methanol in mobile phase B, the initial ratio of mobile phase B compared to mobile phase A, and gradient slope.

During preliminary experiments, we observed an unknown degradation impurity, which eluted just before impurity C. This impurity appears above the reporting threshold level in the sample solution spiked with all nine impurities of ropinirole if exposed to room temperature for at least 24 h. In addition, an unknown impurity, which eluted just after impurity H, was present in the sample of ropinirole hydrochloride. Both impurities were taken into account during all further steps of method development.

### 2.4. Critical Method Attributes and Critical Method Parameters

Our goal was to develop a method able to effectively separate all nine process-related impurities, an unknown degradation impurity, and an unknown impurity present in the sample of ropinirole hydrochloride from ropinirole and from each other. According to the AQbD approach, critical method attributes (CMAs) need to be monitored because they represent the responses that indicate the method performance. Based on the preliminary experiments, the selected CMAs were the tailing factor of ropinirole (T), resolution between impurity A and ropinirole (R1), resolution between impurities E and H (R2), resolution between impurity H and the unknown impurity present in the sample (R3), resolution between impurities G and B (R4), and resolution between the unknown degradation impurity and impurity C (R5).

CMAs are influenced by various factors called critical method parameters (CMPs), which have to be controlled to maintain the proper method performance. Some CMPs were already identified during preliminary experiments; however, their effects on CMAs could not be evaluated through the method scouting experiments performed using OFAT. Nevertheless, the method scouting experiments provided enough information to set the intervals for the factor ranges that needed to be investigated. Finally, six factors were recognized as CMPs and the factor ranges were the following: buffer molarity (10–40 mM), buffer pH (2.0–3.0), column temperature (35–55 °C), the ratio of methanol in mobile phase B (20–40%), the initial ratio of mobile phase B (5–10%), and the gradient slope (0.8–2.4%/min).

### 2.5. Screening Design

Due to a large number of parameters involved in the method being developed, a systematic approach to method development was necessary. Design of experiments (DoE) presents the most important part of AQbD because it makes it possible to obtain a systematically maximum amount of data with a minimum number of experiments performed. Depending on the complexity of the method being developed, DoE may consist of more than one step. The first step is usually a screening design. Screening designs make it possible to study each factor at two levels. The relationship between factors and responses is described by a linear equation for each CMA. For example, for the tailing factor the relation can be written as:

*y*_T_ = *b*_0_ + *b*_1_*x*_1_ + *b*_2_*x*_2_ + *b*_3_*x*_3_ + *b*_4_*x*_4_ + *b*_5_*x*_5_ + *b*_6_*x*_6_ + residuals (1)

where *y*_T_ represents the response measured, *x*_1_, ..., *x*_6_ are selected factors, *b*_0_ is the intercept, and *b*_1_, ..., *b*_6_ are the coefficients for the linear terms.

The screening of six factors according to a full factorial design would require 64 (2^6^) experiments if each factor were investigated at two levels to cover the entire experimental domain. The screening of factors for a method for determining impurities of ropinirole was performed according to a fractional factorial (resolution IV) design. The experimental design was created using MODDE Pro 11.0 software (Umetrics, Umeå, Sweden). In this case, only 16 (2^6−2^ = 2^4^) experiments (with an additional three experiments in the center point) were performed in order to investigate the effects of six factors on the selected CMAs. The experimental design with the responses measured is presented in Table 1.

The aim of the screening design was to determine the effects of factors on each CMA. To this end, the experimental results were statistically analyzed using MODDE software. A multiple linear regression (MLR) method was applied to establish the factor-response relationship; that is, to establish the mathematical model for each response measured. Analysis of variance (ANOVA) was applied to statistically validate the models.

According to the calculated values of statistical parameters (presented in Table 2), the models turned out to fit the experimental data well and show good prediction abilities.

**Figure 3:**
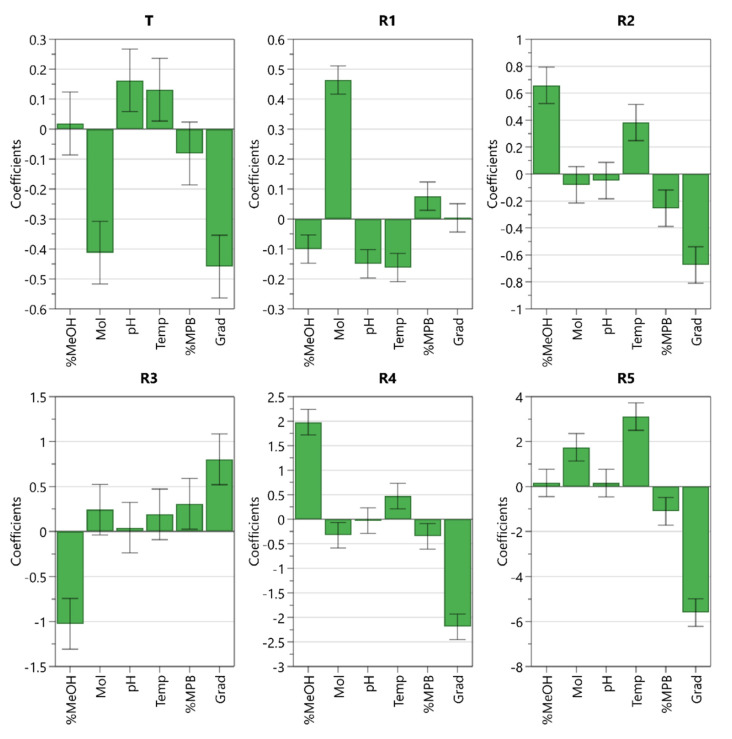
Coefficient plot for models created using fractional factorial (resolution IV) screening design including coefficients and confidence intervals.

The coefficient plot is presented in Figure 3. It can be observed that buffer molarity has very high influence only on the tailing factor (T) and resolution between impurity A and ropinirole (R1). Because higher buffer molarity decreases the tailing factor and increases the resolution, this factor can be set to its upper level investigated.

Other significant factors were the ratio of methanol in mobile phase B, gradient slope, and column temperature. The ratio of methanol in mobile phase B increases the resolutions between impurities E and H (R2) and between impurities G and B (R4), but it decreases the resolution between impurity H and the unknown impurity present in the sample (R3). An opposite effect on these separations was observed with the gradient slope. In addition, a narrower gradient slope lowers the tailing factor (T) and significantly impairs the resolution between unknown degradation impurity and impurity C (R5). Column temperature has a high influence only on the resolution between unknown degradation impurity and impurity C (R5).

The initial ratio of mobile phase B did not turn out to be a significant factor, nor did buffer pH, which negatively affected only the resolution between impurity A and ropinirole (R1), and thus this factor can be set to its lowest level investigated.

Already while performing experiments according to our screening design, we observed that the retention times of impurity C and unknown degradation impurity varied more intensively compared to those of other impurities. Thus, the relative retention time of impurity C according to ropinirole varied from 1.54 to 2.80, and the relative retention time of unknown degradation impurity varied from 1.54 to 2.61. In six experiments performed, these two impurities inverted the elution order, which means that unknown degradation impurity eluted after impurity C (see Table 1, where six responses of R5 have negative values). This was taken into account when setting the criteria for CMAs. The thresholds of acceptable values for all critical resolutions were set to NLT 1.8, except in the case when the unknown degradation impurity eluted after impurity C, where we used a negative precursor instead, and thus the criterion was set to NMT −1.8. The criterion for the tailing factor was set to NMT 3.2.

Due to the good prediction abilities of the models established with screening design, optimal combinations of the CMPs investigated were determined, taking into account the CMA criteria. The first optimal combination is a buffer molarity of 40 mM, buffer pH of 2.0, initial ratio of mobile phase B of 6%, ratio of methanol in mobile phase B of 38%, column temperature of 55 °C, and gradient slope of 2.0%/min, and it applies for the case when an unknown degradation impurity elutes before impurity C. The second optimal combination is a buffer molarity of 40 mM, buffer pH of 2.0, initial ratio of mobile phase B of 6%, ratio of methanol in mobile phase B of 40%, column temperature of 42 °C, and gradient slope of 2.4%/min, and it applies for the case when an unknown degradation impurity elutes after impurity C.

The two optimal combinations share the same values for buffer molarity, buffer pH, and initial ratio of mobile phase B. These results correspond to the observed effects presented in the coefficient plot (Figure 3) because these three factors turn out to be less significant. Therefore, we decided to set these values as constant for further method optimization.

### 2.6. Method Optimization and Robustness Study

In the method optimization step, three factors (column temperature, ratio of methanol in mobile phase B, and gradient slope) and their interactions should be investigated. To this end, a response-surface design was applied because it allows us to study each factor at three levels at least. Here, the relationship between factors and responses is described by a quadratic polynomial, which includes factor interactions. For example, for the tailing factor the relation can be written as:

*y*_T_ = *b*_0_ + *b*_1_*x*_1_ + *b*_2_*x*_2_ + *b*_3_*x*_3_ + *b*_11_*x*_1_^2^ + *b*_22_*x*_2_^2^ + *b*_33_*x*_3_^2^ + *b*_12_*x*_1_*x*_2_ + *b*_13_*x*_1_*x*_3_ + *b*_23_*x*_2_*x*_3_ + *b*_123_*x*_1_*x*_2_*x*_3_ + residuals (2)

where *y*_T_ represents the response measured, *x*_1_, *x*_2_, and *x*_3_ are the selected factors, *b*_0_ is the constant term (intercept), *b*_1_, *b*_2_, and *b*_3_ are the coefficients for the linear terms, *b*_12_, *b*_13_, *b*_23_, and *b*_123_ are the coefficients for the interaction terms, and *b*_11_, *b*_22_, and *b*_33_ are the coefficients for the quadratic terms. The model curvature is described by quadratic terms.

For DoE optimization of a method for determining impurities of ropinirole, the factor ranges of the three highly significant factors were reset in order to cover the optimal values determined with screening design: column temperature (40–57 °C), the ratio of methanol in mobile phase B (32–46%), and gradient slope (1.6–2.8%/min).

Fourteen experiments were carried out according to a central composite face-centered response-surface design with three additional experiments in the center point, and in each experiment the six responses (CMAs) were measured. The experimental design with the responses measured is presented in Table 3.

The mathematical models for each response observed were fitted on the experimental data using an MLR method. The experimental results were analyzed in detail, and non-significant factor terms were excluded from the model. ANOVA was applied to statistically validate the models. All models were proven statistically significant (*p*-value < 0.05). The coefficients of determination (presented in Table 4) show that the models fit the experimental data adequately with non-significant lack-of-fit and indicate good prediction abilities. In addition, a negligible pure error for all models was determined through replicated center point experiments.

In order to examine the method performance in detail, the criteria for CMAs that were already defined during method screening step were taken into account (NMT 3.2 for the tailing factor of ropinirole, and NLT 1.8 for all critical resolutions, except for NMT −1.8 for elution of the unknown degradation impurity after impurity C). A graphic presentation of the dependence of each CMA on the CMPs investigated is called a contour plot. When more than one CMA is monitored, the contour plots of each CMA are overlapped and the sweet spot plot is obtained. The sweet spot plot presents the areas where all CMA criteria are met and where one or more CMA criteria are not met.

**Figure 4:**
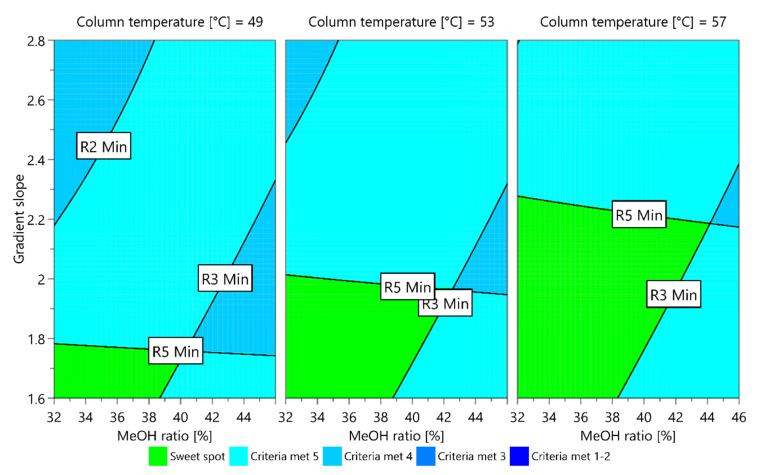
Sweet spot plot obtained for a central composite face-centered design MLR fitted model when unknown degradation impurity is eluting before impurity C. The green field is a sweet spot where all CMA criteria are met.

**Figure 5:**
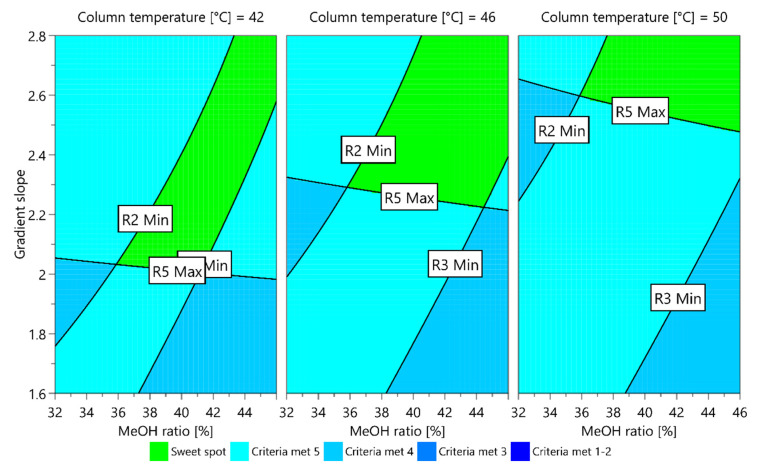
Sweet spot plot obtained for a central composite face-centered design MLR fitted model when unknown degradation impurity is eluting after impurity C. The green field is a sweet spot where all CMA criteria are met.

For the established models, two sweet spot plots were obtained (presented in Figure 4 and Figure 5) with respect to the elution order of the unknown degradation impurity and impurity C using the MODDE integrated sweet spot analysis tool. In each, the sweet spot is presented in green, representing a field where all CMA criteria are met, whereas the light blue represents a field where one criterion is not met.

From the plot presented in Figure 4, it can be seen that a wider sweet spot can be reached if using a higher column temperature. On the other hand, as presented in Figure 5, at a lower column temperature the variation limits in the ratio of methanol in mobile phase B are narrower compared to the ones at a higher column temperature, whereas the opposite was observed for the gradient slope.

Up to this part of the work presented, it can be observed that the method development no longer leads to one set point of method parameters, but the method performance can be prescribed within a certain range of CMPs. However, if the analysis is performed right on the edge of the sweet spot, there will be only a 50% chance that the certain CMA criteria are met. This is because the sweet spot does not take into account possible deviations arising from measurements of factors when performing experiments according to the experimental design as well as model uncertainty.

The purpose of AQbD method development is to ensure that the method will be able to provide accurate and reliable results. To this end, a Monte Carlo simulation method was applied in order to calculate at each point in the sweet spot the risk of failing to meet the CMA criteria. These calculations resulted in a robust area, where the CMA criteria are met with 99% probability, a so-called design space or method operable design region (MODR).

**Figure 6:**
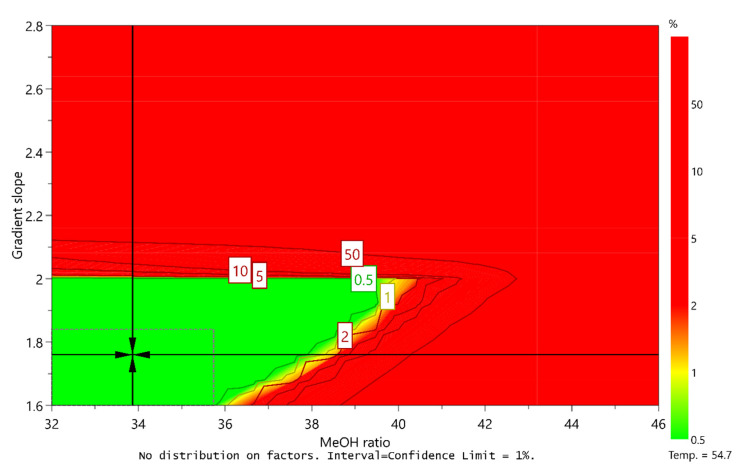
Design space for a central composite face-centered design MLR fitted model when unknown degradation impurity is eluting before impurity C by plotting the ratio of methanol in MP B versus gradient slope at a column temperature of 54.7 °C. Predicted optimal conditions of CMPs are marked with a cross and the defined robustness region is marked with a dashed line.

**Figure 7:**
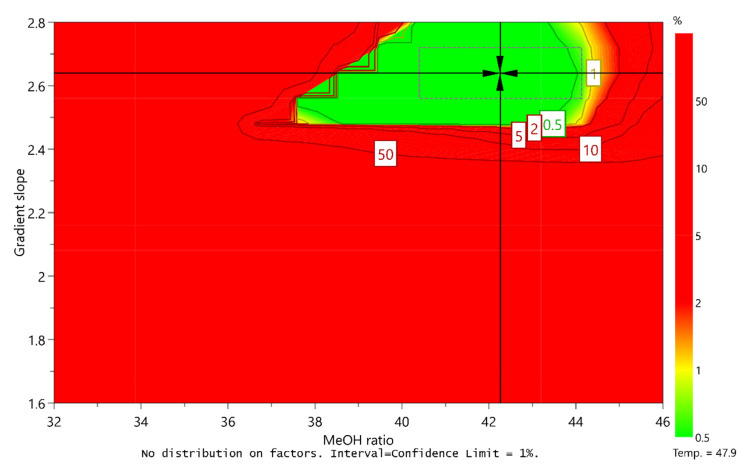
Design space for a central composite face-centered design MLR fitted model when unknown degradation impurity is eluting after impurity C by plotting the ratio of methanol in MP B versus gradient slope at a column temperature of 47.9 °C. Predicted optimal conditions of CMPs are marked with a cross and the defined robustness region is marked with a dashed line.

With respect to different criteria for resolution between unknown degradation impurity and impurity C, two design spaces were obtained (presented in Figure 6 and Figure 7). For each, the range of robustness was defined, and the optimal conditions of CMPs predicted using MODDE were the following:

- Optimal conditions 1 (unknown degradation impurity elutes before impurity C):

Column temperature: 54.7 °C (robust from 52.5 to 57.0 °C)
Ratio of methanol in mobile phase B: 34.0% (robust from 32.0 to 35.7%)
Gradient slope: 1.76%/min (robust from 1.60 to 1.84%/min)

- Optimal conditions 2 (unknown degradation impurity elutes after impurity C):

Column temperature: 47.9 °C (robust from 46.8 to 49.0 °C)
Ratio of methanol in mobile phase B: 42.0% (robust from 40.4 to 44.0%)
Gradient slope: 2.64%/min (robust from 2.56 to 2.72%/min)

**Figure 8:**
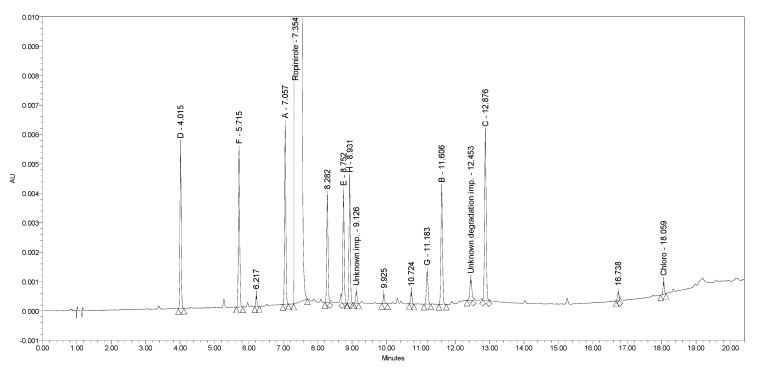
A chromatogram of ropinirole hydrochloride at a concentration of 0.5 mg/mL spiked with nine process-related impurities at the 0.2% level, including unknown impurity present in the sample (t_R_ ≈ 9 min) and unknown degradation impurity (t_R_ ≈ 13 min), obtained using chromatographic conditions 1.

**Figure 9:**
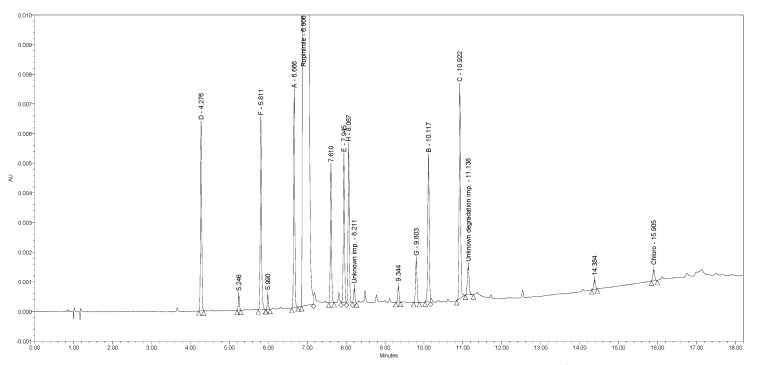
A chromatogram of ropinirole hydrochloride at a concentration of 0.5 mg/mL spiked with nine process-related impurities at the 0.2% level, including unknown impurity present in the sample (t_R_ ≈ 8 min) and unknown degradation impurity (t_R_ ≈ 11 min), obtained using chromatographic conditions 2.

Using the MODDE-established statistical models, a prediction of responses was made for each of the two proposed optimal conditions of CMPs. In order to confirm the validity of the statistical models, experimental confirmation runs were performed. The responses measured (presented in Table 5) match well with the model-predicted values. The final chromatographic conditions for the UHPLC method developed are presented in Table 6. The chromatograms obtained with these conditions are presented in Figure 8 and Figure 9.

Applying the AQbD approach, two method robustness regions were obtained. This means that the analyses can be performed using parameter settings of either the first or second method. Based on a wider robustness area (approximately three times wider), we used optimal chromatographic conditions 1 to perform method validation. In addition, method robustness region 1 was also confirmed experimentally by performing 8 (= 2^3^) experimental runs according to a full factorial screening design. The CMA criteria were confirmed in each experimental run.

### 2.7. Method Validation

The main work of our research was to develop and optimize the UHPLC method presented. In order to confirm that the method (UHPLC chromatographic conditions 1) is suitable for performing analyses in this early development stage the main validation parameters (precision, accuracy, linearity, limit of detection (LOD), and limit of quantification (LOQ)) were tested according to the ICH Q2(R1) [48]. In the late development stages or production of active pharmaceutical ingredient, when method is used for GMP analyses of registration, clinical or production batches, the full method validation is obligatory.

#### 2.7.1. Precision

The precision of the method was tested by injecting six replicates of sample solution of the drug substance. Because the ropinirole hydrochloride sample did not contain any specified impurity above the reporting limit, Ph. Eur. impurities A–H and chloro impurity were spiked to the sample at the 0.15% level (specification limit according to the Ph. Eur. monograph). The results obtained for the six replicates yielded the following intra-day RSD values for each impurity content: 0.55% for impurity A, 0.96% for impurity B, 0.76% for impurity C, 0.83% for impurity D, 1.23% for impurity E, 0.79% for impurity F, 1.04% for impurity G, 0.98% for impurity H, and 0.85% for chloro impurity. The results confirm the suitable precision of the method.

#### 2.7.2. Accuracy

The accuracy of the method was tested by injecting three sample solutions spiked with stock solution of each impurity investigated at three different concentration levels (LOQ, specification limit, and 120% of specification limit) in one replicate. The results of recoveries (presented in Table 7) confirm that the method is accurate.

#### 2.7.3. Linearity

The linearity of the method was tested in one replicate from LOD to 0.50% of the concentration of ropinirole in the sample solution. The stock standard solution was prepared with a concentration of approximately 0.25 mg/mL and was further diluted to obtain six solutions of ropinirole with concentrations in the range 0.025–0.9 µg/mL (0.005–0.18%) and one solution with a concentration of 2.5 µg/mL (0.50%). A value of >0.9999 for the correlation coefficient demonstrated that the method is linear in the concentration range tested.

#### 2.7.4. LOD and LOQ

LOD was determined by one injection of 0.025 µg/mL (0.005%) solution of ropinirole. The signal-to-noise ratio obtained was 16.4. LOQ was determined by six replicate injections of 0.075 µg/mL (0.015%) solution of ropinirole. The average signal-to-noise ratio was 49.9, with the RSD of the ropinirole peak area being 1.0%. The determined LOD and LOQ were 0.005% and 0.015%.

## 3. Materials and Methods

### 3.1. Reagents, Materials and Standards

Acetonitrile HPLC gradient grade (J.T. Baker, Radnor, PA, USA), methanol (ultra) gradient HPLC grade (J.T. Baker), ammonium dihydrogen phosphate for analysis (Merck, Darmstadt, Germany), and purified water were used to prepare mobile phases and solvents. Ortho-phosphoric acid 85% for analysis (Merck) was used to adjust the pH of the mobile phase. The prepared mobile phases were filtered through an Omnipore^TM^ hydrophilic PTFE membrane filter (⌀ = 47 mm) with a pore size of 0.1 μm (Merck).

Ropinirole hydrochloride reference standard, ropinirole hydrochloride drug substance, and standard of Ph. Eur. impurity A were obtained from EDQM (Strasbourg, France). Standards of Ph. Eur. impurities B, C, D, E, G, and H were obtained from TLC Pharmaceutical Standards Ltd. (Newmarket, ON, Canada). The standard of Ph. Eur. impurity F was obtained from Toronto Research Chemicals (North York, ON, Canada). The chloro impurity standard was obtained from Clearsynth (Mumbai, India).

### 3.2. Diluent, Mobile Phase, Column, and Chromatographic Conditions

Mobile phase A was an aqueous phase of 40 mmol/L ammonium phosphate buffer solution, adjusted to pH 2.0 with orthophosphoric acid. Mobile phase B consisted of acetonitrile and methanol in a ratio of 66/34% (*v/v*). For preparation of blank solution, mobile phase A and mobile phase B were mixed in a ratio of 80/20% (*v/v*). Separation was performed on an Acquity UPLC BEH C8 1.7 μm, 150 × 2.1 mm column (Waters Corporation). The column temperature was maintained at 54.7 °C. Flow rate was set to 0.4 mL/min. Gradient conditions were: mobile phase B 6% for 0.5 min, then 6–28% for 13.0 min, then 28–60% for 6.4 min, and return to starting conditions for 0.5 min. Detection wavelength was 250 nm. Injection volume was 2 µL. For DoE purposes, buffer molarity, buffer pH, and initial ratio of mobile phase B were varied as presented in Table 1, and column temperature, the ratio of methanol in mobile phase B, and gradient slope were varied as presented in Table 1 andTable 3.

### 3.3. Equipment and Software

The experiments were performed on a Waters Acquity UPLC H-class system consisting of a quaternary solvent delivery pump, an auto sampler, a column manager, and a photo diode array (PDA) detector (Waters Corporation).

Instrument control and data acquisition were performed using Empower 3 software from Waters (Waters Corporation). Creation of the experimental design and statistical analysis of results were performed using MODDE Pro 11.0 software (Umetrics). The p*K_a_* curves for the nine analytes investigated were calculated using MarvinSketch 17.28.0 software (ChemAxon).

### 3.4. Preparation of Solutions

#### 3.4.1. Preparation of Standard Solution

We accurately weighed 28.5 mg of ropinirole hydrochloride reference standard (corresponding to 25 mg of ropinirole) into a 100 mL volumetric flask. The substance was dissolved and diluted to volume with mobile phase B. Working standard solution was prepared by diluting 1.0 mL of the stock standard solution to 100 mL with the diluent to obtain a 0.002 mg/mL concentration of ropinirole.

#### 3.4.2. Preparation of Sample Solution

We accurately weighed 28.5 mg of ropinirole hydrochloride drug substance into a 50 mL volumetric flask. The substance was dissolved in 10.0 mL of mobile phase B and diluted to volume with mobile phase A.

#### 3.4.3. Preparation of Impurity Solutions

Solutions of impurities were prepared separately. We accurately weighed 2.5 mg of impurity into a 50 mL volumetric flask. The impurity was dissolved in 12.5 mL of mobile phase B. The solution was further diluted to volume with mobile phase A to obtain a stock solution with a concentration of 0.05 mg/mL. Then 1.0 mL of the stock solution was diluted to 20 mL in a volumetric flask, 3.75 mL of mobile phase B was added, and this was diluted to volume with mobile phase A to obtain a solution at approximately the 0.5% level.

#### 3.4.4. Preparation of Spiked Solution for Method Optimization

We accurately weighed 28.5 mg of ropinirole hydrochloride drug substance into a 50 mL volumetric flask. The substance was dissolved in 7.75 mL of mobile phase B. Then 1.0 mL of the stock solution of each impurity was added. The solution was further diluted to volume with mobile phase A. The resulting spiked sample solution contained each impurity at the 0.2% level.

## 4. Conclusions

This work presented the development of a new UHPLC method for determining nine process-related impurities of ropinirole hydrochloride applying the AQbD approach. The current pharmacopeial method was not able to efficiently separate impurities E and H and impurities B and G, and therefore the accurate quantitative determination of these four impurities was impossible. Moreover, the pharmacopeial method prescribes an HPLC separation on a 250 mm long column, resulting in a run time of 75 min, which is not in line with current trends in analytical chemistry and contradicts the principles of green chemistry.

Applying the AQbD approach and using advanced UHPLC technology, we overcame the separation obstacles, and we also improved the pharmacopeial method into a more rapid and green one. In fact, the new method requires less than one-third the run time and just over one-tenth the solvent consumption.

At the same time, DoE methodology provided a greater understanding of method performance. Due to the screening step, a smaller number of preliminary experiments was needed because the screening design also made it possible to uncover the still-unknown effects of certain CMPs. Indeed, only 16 experiments were required to obtain detailed information on the effects of each factor on a particular response, which would not be possible if using the OFAT approach. Applying a response-surface design in the method optimization step, two method robustness regions were defined, which to our knowledge is the first case when AQbD method development has resulted in two design spaces. Usually, if one required a shorter analysis run time, less solvent consumption, and so on than the other, this would be the parameter of choice. In the case presented, there are no significant differences between the two design spaces, and thus the selection was made based on their sizes; that is, the area of robustness of the first optimum is three times larger compared to the second one. Therefore, the first optimum was selected for method validation.

Finally, the UHPLC method developed was validated in terms of precision, accuracy, linearity, and sensitivity, and it was proven to be fit for its intended purpose. We believe that this study provides the first efficient method for reliable control of process-related impurities in ropinirole hydrochloride and will be thus of significant value to the pharmaceutical industry.

## Figures and Tables

**Table 1:** Fractional factorial (resolution IV) design with the results of measured responses (CMAs).

Exp. no.	Exp.	*x*_1_	*x*_2_	*x*_3_	*x*_4_	*x*_5_	*x*_6_	T	R1	R2	R3	R4	R5
1	N14	10	3	55	40	5	0.8	5.15	0.97	4.08	0.10	9.96	10.97
2	N18	25	2.5	45	30	7.5	1.6	3.40	2.06	1.86	2.97	3.99	0.30
3	N11	40	2	55	20	10	0.8	3.33	2.63	1.72	3.89	4.07	9.17
4	N6	10	3	35	40	5	2.4	3.43	1.37	1.51	2.07	3.87	−8.96
5	N10	10	2	55	40	10	2.4	3.42	1.54	2.37	2.42	5.09	−3.59
6	N13	10	3	55	20	10	0.8	4.54	1.34	1.76	3.56	4.55	6.00
7	N2	10	2	35	40	10	0.8	3.83	1.84	2.75	0.10	8.28	0.99
8	N8	40	3	35	40	10	0.8	3.36	2.48	2.58	0.94	7.62	4.96
9	N4	40	2	35	40	5	2.4	2.48	2.56	1.39	2.50	3.42	−5.67
10	N1	10	2	35	20	5	0.8	4.22	1.91	2.19	1.71	5.47	3.24
11	N3	40	2	35	20	10	2.4	2.55	2.87	0.10	4.41	−0.73	−5.88
12	N19	25	2.5	45	30	7.5	1.6	3.39	2.04	1.89	2.92	4.11	0.10
13	N9	10	2	55	20	5	2.4	3.29	1.59	1.43	3.71	1.64	−2.03
14	N17	25	2.5	45	30	7.5	1.6	3.41	2.06	1.84	3.01	3.95	0.79
15	N12	40	2	55	40	5	0.8	3.61	2.10	3.99	0.10	9.36	14.12
16	N16	40	3	55	40	10	2.4	2.90	2.18	2.14	2.74	4.58	0.10
17	N15	40	3	55	20	5	2.4	2.84	2.19	1.12	4.17	0.88	1.79
18	N7	40	3	35	20	5	0.8	3.66	2.54	1.87	2.36	4.55	6.96
19	N5	10	3	35	20	10	2.4	3.45	1.57	0.10	3.56	0.10	−8.94

Exp. no. = number of experiment; Exp. = name of experiment; *x*_1_ = buffer molarity (Mol); *x*_2_ = buffer pH (pH); *x*_3_ = column temperature (Temp); *x*_4_ = ratio of methanol in mobile phase B (%MeOH); *x*_5_ = initial ratio of mobile phase B (%MPB); *x*_6_ = gradient slope (Grad); T = tailing factor of ropinirole; R1 = resolution between impurity A and ropinirole; R2 = resolution between impurities E and H; R3 = resolution between impurity H and unknown impurity; R4 = resolution between impurities G and B; R5 = resolution between unknown degradation impurity and impurity C.

**Table 2:** Statistical parameters of mathematical models obtained with screening design.

Model (Response)	*R* ^2^	*R*^2^ Adj.	*Q* ^2^
T	0.940	0.910	0.822
R1	0.980	0.970	0.944
R2	0.960	0.940	0.879
R3	0.904	0.855	0.746
R4	0.982	0.972	0.952
R5	0.979	0.969	0.944

*R*^2^ = coefficient of determination; *R*^2^ Adj. = adjusted coefficient of determination; *Q*^2^ = predicted coefficient of determination.

**Table 3:** Central composite face-centered design and the results of measured responses (CMAs).

Exp. no.	Exp.	*x*_1_	*x*_2_	*x*_3_	T	R1	R2	R3	R4	R5
1	N14	48.5	39	2.8	2.56	2.28	1.82	2.68	3.77	−3.33
2	N1	40	32	1.6	2.99	2.52	1.85	2.53	4.17	0.01
3	N9	48.5	32	2.2	2.80	2.36	1.76	3.04	3.35	−0.04
4	N10	48.5	46	2.2	2.78	2.21	2.70	1.64	6.26	−1.01
5	N7	57	32	2.8	2.65	2.18	1.81	3.04	2.91	0.01
6	N16	48.5	39	2.2	2.79	2.29	2.20	2.40	4.72	−0.89
7	N8	57	46	2.8	2.63	2.05	2.66	1.95	5.64	−0.96
8	N13	48.5	39	1.6	3.06	2.29	2.70	1.83	6.15	2.55
9	N3	57	32	1.6	3.15	2.21	2.60	2.56	5.08	5.36
10	N17	48.5	39	2.2	2.78	2.30	2.21	2.34	4.75	−1.08
11	N11	40	39	2.2	2.66	2.43	1.76	2.35	4.09	−3.58
12	N6	40	46	2.8	2.46	2.33	1.91	1.86	4.75	−5.85
13	N2	40	46	1.6	2.94	2.34	2.85	0.07	7.24	0.01
14	N15	48.5	39	2.2	2.78	2.30	2.18	2.48	4.64	−0.13
15	N5	40	32	2.8	2.49	2.46	0.91	2.93	1.60	−5.46
16	N4	57	46	1.6	3.11	2.04	3.53	1.11	7.82	5.30
17	N12	57	39	2.2	2.84	2.14	2.56	2.28	5.14	1.67

Exp. no. = number of experiment; Exp. = name of experiment; *x*_1_ = column temperature; *x*_2_ = ratio of methanol in mobile phase B; *x*_3_ = gradient slope; T = tailing factor of ropinirole; R1 = resolution between impurity A and ropinirole; R2 = resolution between impurities E and H; R3 = resolution between impurity H and unknown impurity; R4 = resolution between impurities G and B; R5 = resolution between unknown degradation impurity and impurity C.

**Table 4:** Statistical parameters of mathematical models obtained with response-surface design.

Model (Response)	*p*-Value (Regression)	*R* ^2^	*R*^2^ Adj.	*Q* ^2^	*p*-Value (Lack-of-Fit)
T	1.5465 × 10^−13^	0.999	0.999	0.998	0.421
R1	4.6135 × 10^−12^	0.999	0.999	0.997	0.785
R2	1.2111 × 10^−12^	1.000	0.999	0.997	0.466
R3	6.7141 × 10^−6^	0.949	0.918	0.782	0.084
R4	1.3331 × 10^−12^	1.000	0.999	0.998	0.770
R5	1.2291 × 10^−11^	0.993	0.991	0.988	0.957

*R*^2^ = coefficient of determination; *R*^2^ Adj. = adjusted coefficient of determination; *Q*^2^ = predicted coefficient of determination.

**Table 5:** Statistical model prediction for responses at optimal conditions of CMPs and experimental results obtained in a confirmation run.

	Optimal Conditions 1		Optimal Conditions 2	
CMA (Response)	Predicted	Measured	Predicted	Measured
T	3.05	3.05	2.61	2.68
R1	2.24	2.22	2.26	2.25
R2	2.49	2.41	2.11	2.05
R3	2.63	2.65	2.33	2.38
R4	4.96	4.84	4.64	4.53
R5	3.67	4.25	-2.92	-2.87

**Table 6:** Final chromatographic conditions for the UHPLC method developed.

	Chromatographic Conditions 1			Chromatographic Conditions 2		
Mobile phase A	40 mM ammonium dihydrogen phosphate (pH = 2.0)					
Mobile phase B	acetonitrile/methanol = 66/34% (*v/v*)			acetonitrile/methanol = 58/42% (*v/v*)		
Column	Acquity UPLC BEH C8 1.7 μm, 150 × 2.1 mm					
Column temperature	54.7 °C			47.9 °C		
Flow rate	0.4 mL/min					
Gradient	Time (min)	%A	%B	Time (min)	%A	%B
	0.0	94	6	0.0	94	6
	0.5	94	6	0.5	94	6
	13.5	72	28	13.5	61	39
	19.9	40	60	17.7	40	60
	20.4	94	6	18.2	94	6
Detection	spectrophotometer at 250 nm					
Injection volume	2 μL					

**Table 7:** Results of recoveries of ropinirole impurities investigated at three concentration levels.

Impurity	Concentration Level (%)	Added Conc. (µg/mL)	Found Conc. (µg/mL)	Recovery (%)
Ph. Eur. impurity A	0.05	0.2633	0.2747	104.33
	0.15	0.7899	0.8252	104.47
	0.18	0.9479	0.9923	104.68
Ph. Eur. impurity B	0.05	0.2599	0.2685	103.32
	0.15	0.7797	0.806	103.37
	0.18	0.9356	0.9766	104.38
Ph. Eur. impurity C	0.05	0.2625	0.2687	102.35
	0.15	0.7875	0.808	102.6
	0.18	0.945	0.9709	102.74
Ph. Eur. impurity D	0.05	0.2519	0.2613	103.75
	0.15	0.7556	0.7853	103.93
	0.18	0.9068	0.9435	104.05
Ph. Eur. impurity E	0.05	0.2521	0.2578	102.27
	0.15	0.7564	0.7829	103.51
	0.18	0.9076	0.947	104.34
Ph. Eur. impurity F	0.05	0.2476	0.2606	105.22
	0.15	0.7429	0.7782	104.74
	0.18	0.8915	0.9323	104.57
Ph. Eur. impurity G	0.05	0.2427	0.2437	100.43
	0.15	0.7281	0.7446	102.27
	0.18	0.8737	0.8992	102.91
Ph. Eur. impurity H	0.05	0.2482	0.2554	102.88
	0.15	0.7447	0.778	104.46
	0.18	0.8937	0.9339	104.5
Chloro impurity	0.05	0.2476	0.2713	109.57
	0.15	0.7429	0.7871	105.95
	0.18	0.8915	0.9301	104.33
